# How Technology Advances Research and Practice in Autism Spectrum Disorder: A Narrative Review on Early Detection, Subtype Stratification, and Intervention

**DOI:** 10.3390/brainsci15080890

**Published:** 2025-08-21

**Authors:** Ziqian Shen, Chi-Lin Yu

**Affiliations:** 1 Department of Psychology, University of Michigan, Ann Arbor, MI 48019, USA; ziqshen@umich.edu; 2 Department of Psychology, Oklahoma State University, Stillwater, OK 74078, USA

**Keywords:** autism spectrum disorders, technology, artificial intelligence, early detection and diagnosis, telehealth

## Abstract

While technology has influenced today’s society in many aspects, how does it advance research and practice in the field of autism spectrum disorder (ASD)? In this article, we provide a narrative review of how technology enhances early detection, subtype stratification, and intervention of ASD through advancements in both hardware and software, including neuroimaging, telehealth, and artificial intelligence. Furthermore, given that technology has become an intrinsic part of humans’ daily lives, we discuss how technology can be considered more broadly as a sociocultural context for individuals with ASD in future assessments, diagnoses, and research.

## 1. Introduction

Technological advances influence many aspects of our lives. Smartphones are used every day, and screen media is ubiquitous across our daily lives. We increasingly rely on smart assistants, such as smart speakers, for tasks like checking the weather. We talk to conversational AI tools, such as ChatGPT, to ask questions and for information. Beyond daily uses, technological advances are increasingly contributing to brain and behavioral sciences. This review aims to provide a narrative summary of how technological advancements contribute to the field of autism spectrum disorder (ASD) research and practice, further identifying the current gaps in the field.

ASD is a common neurodevelopmental disorder affecting more than 1% of children worldwide [1,2]. ASD is characterized by early-onset atypical characteristics in social interaction and communication and restricted, repetitive patterns of interests and behaviors. Because of its high prevalence, researchers across developmental psychopathology and medical sciences have been studying epidemiology, underlying mechanisms, and theoretical frameworks of ASD.

Do technological advances influence the field of ASD? The present narrative review discusses the intersection of technological advances and research on ASD, focusing on two critical aspects. The first and primary aspect, “Technology as a Tool,” reviews how technology improves ASD research by enhancing early detection, improving subtype stratification, and supporting intervention of ASD and, thus, technology can be used as a powerful tool to study ASD.

The second and subsidiary aspect, which leans more toward commentary, “Technology as a Context”, discusses how technology, deeply connected to one’s sociocultural context, could be incorporated into our understanding of ASD. For instance, screen media, video gaming, and social networking sites have become intrinsic features in the daily lives of all individuals, including those with ASD [3]. Thus, how these changes influence their behaviors and, consequently, our knowledge of ASD could be considered—that is, viewing technology as a key sociocultural context for ASD.

Accordingly, the present narrative review is divided into two sections: (1) first and primary, on reviewing the use of technology as a tool in the field of ASD (i.e., technology as a tool) and (2) second and subsidiary, on discussing the importance of considering technology as a contextual influence on ASD (i.e., technology as a context). The article ends by highlighting future directions for ASD researchers to consider as they incorporate technological advances and ideas into their work.

## 2. Technology as a Tool

In this section, we review how the field of ASD has benefited from using technology as a tool in three domains: early detection, subtype stratification, and intervention. The conclusion is clear in advance: technological advances have been critical in advancing ASD research and delivering ASD services.

We used the following search process for articles included in this section: Google Scholar, PubMed, and Web of Science were used to identify both empirical and review articles related to ASD and technology. The initial search includes a combination of general keywords such as “autism,” “ASD,” “autism spectrum disorder,” and “technology.” For each subsection, additional topic-specific keywords, for example, “early detection,” “diagnosis,” “diagnostic technology,” “subtype,” “intervention,” and “treatment,” were used in combinations with the general keywords to refine the search for relevant peer-reviewed articles. Articles were screened to include empirical or review articles that focused on ASD and provided substantive discussion or evaluation of a technology.

Consistent with a narrative approach, we did not conduct a formal systematic review or meta-analysis; instead, we focused on selecting representative and methodologically influential studies to illustrate major trends, innovations, and challenges across topic areas and technology areas. Because the literature is heterogeneous in technologies, designs, and outcomes and is rapidly evolving, a narrative review was chosen to prioritize conceptual integration and identification of gaps over pooled effect estimation. We also acknowledge that this approach does not cover the entire field of ASD-related technology research, but it provides a focused framework for evaluating focal developments and identifying promising directions for future investigation.

### 2.1. Early Detection

Early detection of ASD is critical, as diagnosis and interventions within the first few years of life at the early stage of the disorder help improve the quality of life in developmental outcomes [4]. However, a formal diagnosis of ASD is behaviorally defined, and existing early screening tools typically rely on questions and observations from parents or medical professionals (e.g., the Modified Checklist for Autism in Toddlers, M-CHAT [5]; Screening tool for Autism in Toddlers and Young Children, STAT [6]; and Autism Diagnostic Observation Schedule, ADOS [7]). As a result, it remains challenging to detect and diagnose ASD earlier than age four [8]. Some children do not receive a diagnosis until later in the preschool years [9], and this is even more prominent for girls, where girls are less likely to be diagnosed with ASD compared to boys at an early age [10].

The prospects may be changing as a result of the advancement of both hardware technology and software technology. Hardware technologies are typically the primary data source leveraged for early detection, whereas software technologies are the analytic approaches typically applied to that source. In what follows, we organize our review by first discussing influential hardware/data-acquisition modalities, including neuroimaging tools, eye-tracking technologies, and modern touchscreen devices, followed by software/analytic approaches, including supervised machine learning and large language models.

#### 2.1.1. Neuroimaging Tools

First, existing neuroimaging tools have been proven useful in psychology and medical sciences for the past two decades [11]. Technological advancements, particularly in overcoming the challenges of studying infants’ brains, make neuroimaging more beneficial for developmental science, such as infant magnetic resonance imaging (infant MRI) [12], which has increasingly been used in the field of developmental neuroscience (see [13] for a review). Several prospective longitudinal studies have successfully used neuroimaging to identify biomarkers to help with early detection using various helpful neuroimaging tools. For example, by using structural MRI to longitudinally scan 106 infants at high familial risk of ASD and 42 low-risk infants, Hazlett et al. [14] showed the development of brain surface area, as early as 6 to 12 months, predicted the diagnosis of ASD at 24 months. Another diffusion MRI study included 217 infants at high familial risk for ASD at ages 6, 12, and 24 months and showed that the integrities of white matter tracts during infancy predicted symptoms in children who developed ASD at 24 months [15]. A meta-analysis of functional MRI further demonstrated that resting-state functional MRI data have promising sensitivity and specificity, facilitating ASD detection and diagnosis, even for infants as young as 6 months old [16]. Additionally, electroencephalography (EEG) has been a well-established neuroimaging tool used extensively in ASD research (see [17] for a review). For instance, Dickinson et al. [18], with EEG data from 65 three-month-old infants, 36 with familial risk of ASD and 29 low-risk infants, identified biomarkers in frontal and right temporoparietal connectivity that predict ASD symptoms at age 18 months. Beyond these, many studies also provide similar evidence in the detection of ASD (see [19,20] for recent reviews). Together, these neuroimaging technologies, ranging from EEG to MRI, can be helpful for the early detection of ASD and do so as early as 3 months old.

#### 2.1.2. Eye-Tracking Technologies

Second, new eye-tracking technologies provide convenient and objective assessments for psychologists to diagnose ASD based on eye fixation and eye gazes in infants and young children [21,22]. Eye-tracking tools, such as emotion recognition detection [23] and eye gaze pattern detection [24], have shown effectiveness and reliability in the early detection of ASD risks. For example, Wan et al. [25] conducted a study on applying eye-tracking technology to identify ASD in children. They studied 37 children with ASD and 37 neurotypical children aged 4–6 years and explored their eye fixation times on different areas of the face when viewing a video of a female speaking. They found that participants’ eye fixation time, particularly at the moving mouth and body, could distinguish children with ASD from neurotypical children with more than 85% accuracy. Similarly, Murias et al. [26] asked 25 children with ASD aged 24 to 72 months to watch a video of a speaker giving a child-directed speech. They found that participants’ total gaze fixation time (on the speaker) in the video was associated with their social communication skills, which is one of the clinical measurements in diagnosing ASD. More generally, in a recent large-scale study with 475 16- to 30-month-old children, Jones et al. [27] used eye-tracking technology to identify biomarkers of ASD in their visual engagement by watching videos of social interaction content and found that eye fixation patterns can predict ASD diagnoses with over 70% accuracy. Their eye-tracking technology, EarliPoint, was approved by the U.S. Food and Drug Administration (FDA) as a diagnostic tool for ASD, highlighting a milestone in the clinical practice of early ASD detection in children as young as 16 months [28]. Overall, these new eye-tracking technologies prove promising as a convenient and speedy screening tool to aid professionals in the early detection of ASD.

#### 2.1.3. Touchscreen Devices

Third, the increasingly common use of modern touchscreen devices, which can help capture motor-related information, is helpful for early detection of ASD in children, particularly concerning motor difficulties. For example, Lu et al. [29] analyzed the swipe pattern of young children aged 25–79 months (37 children with ASD and 45 neurotypical children) on an iPad through gameplay. They found that the younger children with ASD (<5 years old) demonstrated faster goal-directed swipes compared to neurotypical children, whereas older children with ASD (>5 years old) showed slower goal-directed swipes. Relatedly, Anzulewicz et al. [30] recruited 36 children with ASD between the ages of 3–6 and 45 age-matched neurotypical counterparts and tested their motor patterns in the gameplay on a touchscreen device (e.g., an iPad). They found that children with ASD use greater force in pressing the screen, faster gestures in gameplay, larger and more distal gestures, and faster screening tapping compared to neurotypical children. These motor patterns have more than 90% accuracy to distinguish children with ASD from neurotypical children. Similarly, Simeoli et al. [31] studied 60 children between the ages of 5 and 10 (30 children with ASD and 30 neurotypical children) on their dragging movements on a touchscreen device (e.g., an Android tablet). By looking at dragging movements and touch data, they found that children with ASD showed low linearity and greater irregularity in their movements. Similarly to touchscreen devices, Bondioli et al. [32] designed a smart toy system that has motion sensors built in to detect repetitive motor movements during free play in children with ASD. They measured the play patterns in 50 preschoolers (25 children with ASD and 25 neurotypical children) aged 3–5 years and reported an overall accuracy rate of more than 90% in detecting the repetitive motor patterns that children with ASD present. In brief, these studies, among many others (see [33,34]), capitalize on the now-typical childhood activity—using a touchscreen device—demonstrating the potential effectiveness of using touchscreen devices as an accessible and child-friendly tool to support the early detection of ASD.

#### 2.1.4. Supervised Machine Learning

Concerning the advancement in software technology, one development that improves the early detection of ASD is supervised machine learning algorithms [35]. Supervised learning algorithms refer to a group of statistical models that can learn from the data and apply the learned information to predict new data [36,37]. Examples include deep neural networks, support vector machines, and tree-based methods. Since the diagnosis of ASD can be viewed as a predictive task (i.e., based on the features of a child, we predict whether he/she has ASD), implementing supervised learning algorithms to establish reliable ASD screening tools for early detection is promising (see [35,38] for recent reviews). For instance, Abbas et al. [39] applied a random forest algorithm [40] on parent-reported questionnaires and home videos of children to predict the diagnostic statuses of a sample of 163 children between 18 and 72 months of age. They found that this approach outperformed other early screening tools (e.g., M-CHAT) in the prediction performance. In fact, their tool [39], officially named Canvas Dx, later became the first FDA-approved diagnostic tool using supervised machine learning algorithms to predict ASD [41]. Similarly, Mazumdar et al. [42] found that naive Bayes classifiers and support vector classifiers could successfully predict a child’s ASD status based on their viewing behaviors while seeing simple images. In a recent systematic review by Ahmed et al. [43], algorithms such as support vector machines, GoogleNet, and ResNet-18 have achieved high accuracy and performance of 99.8%, 93.6%, and 97.6%, respectively, in predicting early diagnosis of ASD. These studies highlight the potential of supervised learning algorithms to improve the effectiveness of early ASD screeners. Note that while this subsection highlights the use of supervised learning algorithms in early ASD detection, unsupervised learning algorithms are helpful (see [20] for a recent review).

#### 2.1.5. Large Language Models

Lastly, natural language processing computational models, such as Bidirectional Encoder Representations from Transformers (BERT) [44], Generative Pre-trained Transformers (GPT), or large language models (LLM) in general, have gained increasingly more attention in the early detection of ASD for the past few years. For example, Zhang et al. [45] used BERT to analyze the audio recordings of 76 children aged 7–16 in their clinical interviews and assessments with a psychiatrist. They concluded that the BERT language model has an accuracy greater than 85% in classifying ASD. Furthermore, Mukherjee et al. [46] used BERT and ChatGPT to analyze parents’ conversations about their children with ASD. These models could accurately label each sentence in dialog with specific behavior categories, such as eye contact or behavioral problems, related to ASD criteria and symptoms. Also, a recent study by Themistocleous et al. [47] used BERT through storytelling to examine linguistic markers (such as grammatical and semantic features) of ASD in 68 children with ASD and 52 typically developing children aged 4–11. They found that the natural language processing model achieved an accuracy of 96% in detecting ASD. Although using natural language processing models in ASD detection is just coming into view, these emerging studies have shed new light on how these models can assist in detecting language and speech patterns related to ASD in young children, hopefully aiding in the early detection of ASD in the near future. Indeed, recent reviews have shown how natural language processing models can be applied to medical, mental health, and other diverse fields, including ASD [48].

#### 2.1.6. Summary

In brief, these advancements in hardware (i.e., neuroimaging tools, eye tracking technologies, and touchscreen devices) and software (i.e., supervised learning algorithms and natural language processing models) have the potential to help improve the early detection of ASD. If appropriate, combining both hardware and software tools can provide even better results for the early detection of ASD [18,49].

However, these technologies have critical limitations. Neuroimaging tools like MRI are costly, time-intensive, and often impractical for routine clinical practice, especially in younger populations [19]; eye-tracking ASD detection, as well as the use of touchscreen devices, are sensitive to sample, stimulus, and calibration differences, making replication and generalization difficult [22,29]; the work of LLM is still preliminary and lacks formal clinical evaluation, and its issues of privacy and potential cultural bias make LLMs not yet ready for routine clinical use [48]. These limitations constrain these tools’ immediate clinical use, but at the same time, they clarify priorities for translation: standardized protocols, validation in diverse populations, and cost-effectiveness and implementation planning. Addressing these issues would be essential to move from promising innovations to reliable tools for everyday use in early ASD detection.

### 2.2. Subtype Stratification

ASD is a complex condition characterized by heterogeneity in symptom severity [50], cognitive functioning [51], biological profiles (see [52]), and developmental trajectories [53]. Effective subtype stratification is needed to understand and address this heterogeneity, as it allows for developmental prediction and assessment of ASD on an individual level [54,55].

Unsupervised machine learning algorithms are particularly beneficial technological advances for subtype stratification [36]. Unlike supervised learning algorithms, which aim to make accurate predictions based on labeled data, unsupervised learning can identify hidden structures and patterns based on the embedded statistical distinctions in unlabeled data without prior assumptions or subjective human input. In the context of ASD, unsupervised learning can help reveal potential underlying subtypes within a group of individuals with ASD based on the statistical properties of their data.

Many studies have successfully used unsupervised learning algorithms to perform subtype stratification of ASD (see [55] for a review). For example, Duffy & Als [56] applied the NbClust algorithm to the EEG signals of 430 children with ASD and identified two statistically different subgroups of ASD, where one of the subgroups demonstrated more Asperger-like traits, and the other subgroup did not. They reported that the NbClust algorithm and EEG coherence serve as an unbiased and objective method of determining the subgroups of ASD in children.

Also, Easson et al. [57] applied k-means clustering on 145 individuals with ASD and 121 neurotypical individuals and identified two subtypes based on the differences in brain connectivity related to behaviors. They showed that the ASD participants in the two subtypes, although different in their brain connectivity, did not differ significantly in their ADOS scores, which indicated that brain connectivity in ASD might reflect unique brain-behavior patterns that could not be easily found without the use of unsupervised learning algorithms.

Moreover, Stevens et al. [58] implemented Gaussian Mixture Models and Hierarchical Clustering on a large sample of 2400 children with ASD (ages 2.66 to 12) from the archived database. They found 16 unique ASD clusters and 5 hierarchical subgroups based on the severity across eight developmental domains, such as language, motor skills, and cognitive function.

Building on this, Gardner-Hoag et al. [59] used a k-means clustering algorithm to analyze data on challenging behaviors reported by clinicians during treatment (i.e., clinicians would record behavioral data in an iPad during treatment sessions whenever they encountered challenging behaviors of ASD, e.g., aggression, tantrums, and noncompliance) in 854 children with ASD ages 18 months to 12 years old. They identified 8 subtypes of ASD based on the differences in their repeated challenging behaviors.

More recently, a study by Litman et al. [60] used a person-centered approach with unsupervised mixture modeling on a large sample of ASD totaling more than 5000 people (aged 4–18 years). They identified four latent subgroups for individuals with ASD: moderate challenges, social/behavioral, mixed ASD with developmental delay, and broadly affected. These ASD subgroups differed in milestone timing, age at diagnosis, and language ability and showed distinct genetic signatures across polygenic scores and rare/de novo variants.

In short, these advancements help researchers and clinicians detect subgroups of ASD early on, even during preverbal ages, and lead to a better understanding of the underlying mechanisms of ASD in research. The findings would assist in providing more individualized support and interventions through treatment tailored to the specific needs of each subtype of individual with ASD. Still, using unsupervised machine learning methods in practice requires caution: subtype solutions are often associated with preprocessing choices and algorithm parameters, stability and generalizability are rarely tested across larger samples, and subtype utility can be limited if subtype patterns do not predict outcomes for a larger population [61]. Overall, while this area of research is still developing and needs more translational considerations, the advances of unsupervised learning algorithms hold significant promise in improving ASD subtype stratification (see [61] for a recent review).

### 2.3. Intervention

Technological advancements have been proven helpful in delivering interventions in the context of ASD. Generally, technology-based interventions have shown high effectiveness in improving ASD symptoms [62,63]. Potentially, this is because of the unique interests of individuals with ASD in technology, coupled with the features of technology (such as clear instructions, reduced distractions, and lower social demands) that align with the difficulties of ASD, making technology-based interventions suitable for this population. Thus, numerous technology-based interventions have been developed, including telehealth therapies, smartphone applications, computer programs, virtual reality, interactive videos, robotics, and artificial intelligence chatbot. Four notable technology-based interventions demonstrating promising results in improving ASD outcomes are highlighted below.

#### 2.3.1. Telehealth Therapies

First, telehealth therapies, accomplished by the high-speed internet, are useful for reaching individuals who fail to seek or receive appropriate health services, as they can overcome the geographical distance barriers between professionals and receivers and provide flexibility in time and location. They are also beneficial in the COVID-19 stay-at-home period, while most communications, including the implementation of therapies, have been transitioned to a remote format [64]. Most of all, along with these practical advantages, telehealth therapies have been found to be equally effective in ASD. For example, Conaughton et al. [65] conducted a randomized controlled trial in 42 children (ages 8–12) to evaluate the efficacy of an online cognitive behavior therapy program in children with ASD and comorbid anxiety. Their results showed that relative to children who did not receive any intervention, children with ASD who received the online intervention demonstrated a reduction in anxiety symptoms. Lindgren et al. [66] assessed the effectiveness of a telehealth functional communication training program in 38 children with ASD. They found a reduction in problem behavior and social communication in children after a 12-week telehealth intervention compared to an in-person control group. Lastly, Hao et al. [67] compared telehealth therapy and traditional face-to-face therapy in two groups of families who had children with ASD, one group participating in person (*N* = 65) and the other group participating via telehealth video therapy (*N* = 40). They showed significant improvements in children’s performance in both groups, with equal effectiveness across two therapy delivery groups. Overall, the flexibility and fidelity of telehealth therapies (see [68,69,70] for recent reviews) make them an excellent fit for individuals with ASD.

#### 2.3.2. Smart Devices and Digital Applications

The second case that deserves mention is the impact of smart devices, primarily smartphones and tablets. These devices have positively affected the field of ASD, mainly because these devices can be coupled with various applications to deliver ASD services. Two main functions of the applications that help individuals with ASD are discussed here: augmentative and alternative communication through speech-generating technologies and behavioral intervention software.

First, the augmentative and alternative communication interventions [71] via smart devices have shown promise in supporting individuals with ASD. For example, a systematic review showed that these smart devices could function as speech-generating devices and evidenced that the individuals with ASD who use these devices can acquire verbal skills quickly [72]. Likewise, An et al. [73] developed an app, available in both iOS and Android versions, called Yuudee, that contains over 400 drawings with corresponding phrases to assist children with ASD in communicating requests and expressing emotions. Their initial findings on 10 children with ASD have demonstrated how this app can effectively train children with ASD. Similarly, dedicated devices, such as Nova Chat, Dyna Vox, and Vantage Light, have also been widely used and evaluated in the field [74,75]. These advances altogether have shown effectiveness in enhancing communication and verbal behavior from children with ASD (see [76,77] for reviews).

Second, a range of digital applications has been developed to address other social and behavioral challenges associated with ASD. For instance, Fletcher-Watson et al. [78] developed an IOS app, FindMe, to train social communication skills in children with ASD. The app provides interactive scenarios to teach children with ASD to attend to people and follow social cues, such as tapping on the character on the screen during a play. Their empirical evidence with 54 children with ASD showed that FindMe delivered the training as effectively as the usual intervention the children received. Furthermore, Whitehouse et al. [79] evaluated an app called Therapy Outcomes By You (TOBY) that focused on the ASD learning curriculum in four developmental areas: visual and auditory understanding, imitation, receptive and expressive language, and social skills. They found that compared to the parallel control group, which only attended therapy, children who attended therapy and utilized TOBY regularly showed an improvement in developmental skills after six months. Overall, the effectiveness of these advances has been reported in various studies [80,81], suggesting the broad potential of using smart devices and digital applications as a valuable tool in the ASD intervention.

#### 2.3.3. Virtual Reality

The third innovative intervention is virtual reality (VR) [82,83] to aid in delivering treatment to individuals with ASD. For example, Cai et al. [84] developed a VR application that assisted in providing autism therapy to children with ASD. They created an interactive virtual dolphinarium, which allows children with ASD to act as dolphin trainers and learn nonverbal communication (e.g., hand gestures) with virtual dolphins. They completed a pilot study on 15 children with ASD and found that children improved their nonverbal communication as long as they worked well with the 3D glasses (although it was not effective when the 3D glasses were too overwhelming). Moreover, Yuan & Ip [85] examined the effectiveness of an immersive VR training program, Cave Automatic Virtual Environment (CAVE), which focused on emotional and social skills development. The CAVE design was a four-sided room, allowing real-life simulations and interactions. Their evaluations with 72 children with ASD showed that children who received the VR training improved social interaction (e.g., communicating with neighbors) and adaptation (e.g., flexibility in seat preferences) better than children who did not. Some recent studies even showed that VR is more effective than traditional interventions. For example, Frolli et al. [86] compared the effectiveness of how children with ASD recognize emotions in two groups: VR intervention (e.g., 3D projection of simulations of social scenes with emotions) and therapist intervention (e.g., photos and images that convey a feeling). They found that children in VR intervention learned emotional expression faster than in the therapist intervention. Overall, VR technology shows promising potential (see [87] for a recent review) in creating a safe and supportive virtual environment and interventions tailored to the needs of children with ASD.

#### 2.3.4. Artificial Intelligence Chatbot

Finally, a recent notable advancement is, again, LLM-based artificial intelligence (AI) chatbot technologies, such as ChatGPT, which can provide fast and individualized responses during interaction. By hypothesis, AI can be helpful in the future of ASD in many ways, such as providing emotional support through speech interaction or practicing developmental skills through role-play activities. One study has a first glance at how it can be helpful. Bertacchini et al. [88] combined ChatGPT with Pepper, a humanoid social robot, to generate individualized responses in simulations of social interactions. For instance, Pepper could simulate different interaction scenarios that target individual needs for various domains, such as communication, social skills, and problem-solving. They showed the possibility that such an AI advancement could interact with individuals with ASD to further help train and enhance their communication skills. Another study by Aydemir [89] evaluated the effectiveness of a four-week physical activity intervention in 26 families (13 children with ASD, and 13 typically developing children as a control group, with an average age of 14 years old) using suggestions made by ChatGPT. Parents received training from the researcher on how to properly ask ChatGPT using appropriate commands for suggestions that best fit their child. The result showed that children with ASD experienced a significant increase in their physical activity levels, and the parents are satisfied with the ChatGPT-delivered intervention. This study further shows potential for caregivers to utilize tools like ChatGPT for information tailored to their child’s needs as a supplement to their ASD interventions; however, importantly, it does not suggest using AI chatbots to replace human intervention. Overall, as relatively cost-effective, time-saving, and easily accessible, AI chatbots may benefit the field of ASD by providing interventions in social interaction and communication skills, especially in locales that lack resources to offer ASD intervention.

#### 2.3.5. Summary

These technology-based interventions are promising but differ in limitations and clinical feasibility. Telehealth services face issues with accessibility, particularly for families or regions without reliable internet or access to devices [70]. Research on smart devices and digital applications requires more generalizability and application in diverse clinical and classroom settings before we fully adopt it and conclude its effectiveness [72]. VR tools can be limited in their cost and/or discomfort of wearing VR glasses or headsets [83]. Also, AI chatbots may in fact require intensive clinician oversight because of their known issues about privacy, potential bias, and hallucinations [90,91]. In brief, although not without concerns, technology-based interventions can be helpful and effective [62,92] and would benefit individuals with ASD if made widely available.

## 3. Technology as a Context

The previous section summarized how researchers can use technology as a tool to help ASD research across three domains. This section serves as a commentary to discuss how technology might need to be considered more broadly as a context beyond a tool.

To reiterate, using technology has become an intrinsic feature in the daily lives of individuals with and without ASD. Many households have screen media and smart home devices that are regularly used by all individuals, including both children and adults, with and without ASD. For children with ASD, using screen media for activities such as watching videos and playing video games is common and occupies a good amount of time in their daily lives [3,93,94]. Just like typically developing individuals, bedroom media is also one of the most common rituals for individuals with ASD [95]. Similarly, most individuals with ASD use social media, such as social networking sites and instant messaging, to interact with others and engage in closer friend relationships [96]. Overall, technology has been deeply embedded in the sociocultural context of human life, affecting both individuals with ASD and those without.

However, technology’s influences have not yet been widely integrated into ASD research and diagnosis despite its significant influences on people’s daily lives. Take diagnostic screening tools as an example. Researchers have developed many helpful instruments to diagnose ASD [1]. Observation tools, such as the Autism Diagnostic Observation Schedule, Second Edition (ADOS-2 [97]), allow professionals to observe the particular behaviors of the individuals; caregiver interviews, such as the Autism Diagnostic Interview-Revised (ADI-R [98]), allow professionals to understand a more comprehensive developmental history of a child; symptom assessments, such as the Social Communication Questionnaire (SCQ [99]), obtain the severity of symptoms via parent report. These instruments have provided significant contributions to the field, but they also reflect assessments before technology became an essential part of daily life. Could adding the sociocultural context of technology uses in these screening tools benefit the understanding and treatment of the modern-day ASD population? We use the aforementioned three instruments as an example to elaborate this point.

The ADOS-2 uses structured activities and materials to elicit and observe spontaneous social and communicative behaviors relevant to ASD [2]. ADOS-2 has four modules for individuals suspected of ASD based on their age groups, from toddlers to adults. These interactive activities involve using toys and free vs. structured play to demonstrate whether a child or the individual is socially responsive. The range of activities varies from toddlers (e.g., responding to their name), children (e.g., telling a story), to adults (e.g., demonstration tasks). However, these activities are separate from interacting with technology or people via technology, despite its presence in most children’s daily lives. Creating new activities that use common technology (e.g., smartphones and tablets) to prompt behaviors that reflect their daily lives may be useful. For instance, professionals can play an interactive game on a tablet or more engaging games on a virtual reality platform with the child. They can, thus, observe whether children show target behaviors (e.g., facial expression, usual and unusual social responses, joint attention, conversations, or insights) in such interactions involving technology. Similarly, in less structured, more conversational types of tasks, professionals can also use technology-based chat software or social networking sites to generate conversations and interactions with children, aiming to observe whether they have the conversational ability to perform back-and-forth interchange using, for example, text or emoji. Additionally, considering the new sociocultural environments advanced by technology, these subtle edits/additions can help more comprehensively assess the children’s social and conversational abilities across a broader range of contexts. Also, given that children with ASD could act differently when using technology (e.g., interactions with conversational AI technology such as ChatGPT) compared to regular face-to-face interactions, considering technology in ADOS’s interactive activities would help contribute to more precise diagnoses.

Likewise, the SCQ can have a similar update. The SCQ is a screening tool given to caregivers of individuals with ASD to assess the symptoms exhibited not only currently but also early in life [99]. The SCQ includes questions such as “Has she/he ever use socially inappropriate questions or statements?” “Has she/he ever have any interests that preoccupy her/him and might seem odd to other people?” and “Does she/he play any pretend or make-believe games?”. The SCQ may also benefit from adding questions about the conversation happening in the context of technology or adding an extension to make these questions include situations under the context of technology. For instance, revised from the first exemplary question, the new question could be “Has she/he ever use socially inappropriate questions or statements, either in-person or on digital platforms, such as social media or online group chat?” For the second exemplary question, the revised question could highlight the chance that the child shows special interests in some odd applications/software: “Has she/he ever have any interests that preoccupy her/him and might seem odd to other people, including small electronic devices, computer software, and online activities?”. Lastly, “Does she/he play any pretend or make-believe games, including with conversational AI (e.g., Siri, Google, Alexa)?” These twists may help the caregivers to further consider the children’s situations, social interactions, and behaviors more widely, under both the contexts of having technology as well as regular face-to-face context.

Another screening tool is the ADI-R. ADI-R is a semistructured interview for caregivers of individuals at risk of ASD [98]. This assessment includes qualitative questions about abnormalities in two main categories: reciprocal social interaction (e.g., direct gaze, sharing, etc.) and communication (e.g., pointing to express interest, imitating play, etc.). ADI-R provides professionals with a more holistic understanding of the individual’s social interactions with others in everyday contact. The ADI-R contains sample questions, such as asking how children engage and interact with their peers or form friendships. The ADI-R may benefit from additional twists in these questions to better understand children’s interactions by including both technology and humans. For example, these questions could weave in technology by including interactions with technology or observable changes in engagement with AI chatbots or social robots. These modifications and additions help evaluate the context of technology in the present-day world as more daily activities and schools are transitioning to using technology to provide convenience. Furthermore, considering technology is important in today’s society, caregivers might benefit from incorporating technology into their observations. Helpful technology, such as sensory monitors for extreme emotional changes, tracking for behavioral outbursts, or smartphone applications to set routines and schedules, are additional tools for caregivers to reference their observations of individuals at risk of ASD during ADI-R, instead of relying only on their subjective observations or memories.

Taken together, by taking the impacts of technology into account, the existing diagnostic instruments may be enhanced to more comprehensively assess individuals’ behaviors to reflect their daily lives, especially when the children in the current generation are possibly equally familiar with technology as actual toys and equally familiar with interactions using technology as in person. Note that we do not suggest replacing existing activities or items or rendering these instruments solely relevant to technology. Rather, given the importance of technology in our daily lives, considering technology as an everyday context would additionally benefit our understanding of ASD.

Furthermore, considering technology as a context offers researchers a critical lens to examine variability in technology accessibility. Individuals or groups with ASD may experience varying technological access levels based on their specific scenarios. For instance, the socioeconomic status of a family significantly impacts the technology and technical resources available to the child with ASD. Geographic context also plays a role; for example, in many developing countries, ASD diagnosis is less prevalent due to limited screening resources, and access to technology is often constrained. These differences manifest in the various aspects of using technology, including internet access, internet speed, exposure to new information, applying technology in educational resources, and the outcomes of using these technologies over time [100]. One of the most notable examples is the unequal accessibility to telemedicine services among individuals in developing countries, racial and ethnic minority groups, and lower-income households [101]. To address these challenges, future research and practice should explicitly acknowledge these disparities and promote strategies for improving technology accessibility (e.g., developing more affordable technologies), providing healthcare resources (e.g., increasing awareness of ASD), and expanding intervention support (e.g., via affordable telehealth services) as technology use becomes more prominent. Additionally, longitudinal and cross-cultural studies examining the effectiveness of technology-based ASD interventions in resource-limited settings could provide valuable insights; such efforts would not only facilitate access to novel technology but also support the ASD community across global contexts.

Although we have mostly discussed how technology can benefit individuals with ASD, many studies have also documented its potential negative effects, particularly with early and prolonged screen media exposure. Concerns include reduced sleep duration and quality, decreased physical activity, and increased risk of gaming addiction [93]. Some researchers also speculate that such exposure may contribute to diminished attention and deficits in certain cognitive and social abilities (in both individuals with and without ASD). For example, Dong et al. [102] reported that longer screen time may be associated with increased severity of ASD symptoms. Thus, we note the need to balance technology use and, once again, to consider how technology is integrated into individuals’ daily sociocultural contexts to better understand both its positive and negative influences.

Overall, technology has become an essential part of our daily lives and should be considered more carefully in research and diagnostic tools. At the same time, the effects of technology use on individuals with ASD may vary depending on accessibility, and the issues around the accessibility of ASD resources are likely to arise soon, especially because ASD-focused technological resources are becoming more prominent nowadays. Therefore, considering current technology in the research and diagnostic tools of ASD will be critical to understanding ASD more comprehensively at both individual and community levels.

## 4. Conclusions and Future Directions

Although the benefits of technology for individuals with ASD have been increasingly considered recently, uncovering this topic in more detail is essential because of technology’s demonstrable importance to children’s social lives and the growing prevalence of ASD in our world. In this narrative review, we discussed how technological advances influence ASD research from two critical aspects, “Technology as a Tool” and “Technology as a Context.” Two conclusions deserve a special note. First, technology can be a powerful tool for detecting difficulties earlier, identifying subtypes more precisely, and offering interventions more conveniently. Thus, in order to benefit real-world clinical practice, future studies should continue developing more accessible and cost-effective ASD-related tools, validating AI-based screening tools for accuracy, and improving existing technologies for their efficacy. Additionally, future research should also aim to address the challenges in areas that have limited access to the use of technologies like virtual reality and AI tools to promote equity. In the near future, technology is best integrated within a human–technology collaboration model in ASD, where it augments, not replaces, clinician judgment and support, while standards, validation, equity, and effectiveness continue to develop.

Second, technology has not been widely considered as a context, even though it deeply embeds our daily lives. Future research should acknowledge and systematically examine how technology has changed the sociocultural context of individuals with ASD and their interaction with digital environments. These changes may have uniquely transformed experiences and manifested in the context of individuals with ASD. As an immediate, actionable target, developing and incorporating measures of technological use in diagnostic instruments could allow more comprehensive assessments, potentially more accurate diagnoses, and more advanced understanding of ASD within the current sociocultural contexts.

In summary, this narrative review discusses how technology has been used and could be considered further in the field of ASD research and practice. Using technology as a tool can improve the efficacy of the research on ASD and interventions provided to individuals with ASD. Considering technology as a context could offer more comprehensive understanding of ASD. An integrative perspective encompassing both aspects can inform the most about ASD and ASD research.

## Abbreviations

The following abbreviations are used in this manuscript:

ASD	Autism Spectrum Disorder
BERT	Bidirectional Encoder Representations from Transformers
GPT	Generative Pre-trained Transformers
LLM	Large language models
EEG	Electrophysiology
TOBY	Therapy Outcomes by You
VR	Virtual Reality
CAVE	Cave Automatic Virtual Environment
AI	Artificial Intelligence
ADOS-2	Autism Diagnostic Observation Schedule, Second Edition
SCQ	Social Communication Questionnaire
ADI-R	Autism Diagnostic Interview-Revised
FDA	Food and Drug Administration
